# A Rare Primary Pulmonary Chondrosarcoma

**DOI:** 10.7759/cureus.16665

**Published:** 2021-07-27

**Authors:** Siham Nasri, Yassine Amane, Ihsane Alloubi, Narjisse Aichouni, Imane Skiker

**Affiliations:** 1 Radiology, Mohammed Sixth University Hospital, Oujda, MAR; 2 Faculty of Medicine and Pharmacy, Mohammed First University, Oujda, MAR; 3 Thoracic-Cardiac Surgery, Mohammed Sixth University Hospital, Oujda, MAR; 4 Epidemiological Laboratory of Clinical Research and Public Health Faculty of Medicine and Pharmacy, Mohammed First University, Oujda, MAR

**Keywords:** chondrosarcoma, lung, ct scan, cancer, trans thoracic biopsy

## Abstract

Malignant cartilage tumors of the lung are unusual. Differentiation from other benign cartilaginous tumors of the lung can be challenging, thus careful analysis is of the essence. Imaging study is non-specific, surgery remains the main treatment in localized forms. Herein, we report a case of primary pulmonary chondrosarcoma.

## Introduction

A rare tumor is generally defined by the absence of up-to-date clinical or evolutionary data and the absence of specific therapeutic data. A rare lung tumor develops from orthotopic, heterotopic, or hematopoietic tissues. The most common rare lung tumors are in decreasing order of prevalence, carcinoid tumors, sclerosing hemangiomas, myofibroblastic tumors, mucosa-associated lymphoid tissue (MALT) lymphomas, and neuroblastomas. Chondrosarcoma is exceptionally primary to the lung. No frequency data have been published. Herein, we report a new observation in perspective to contribute to the other few similar cases.

## Case presentation

**Figure 1:**
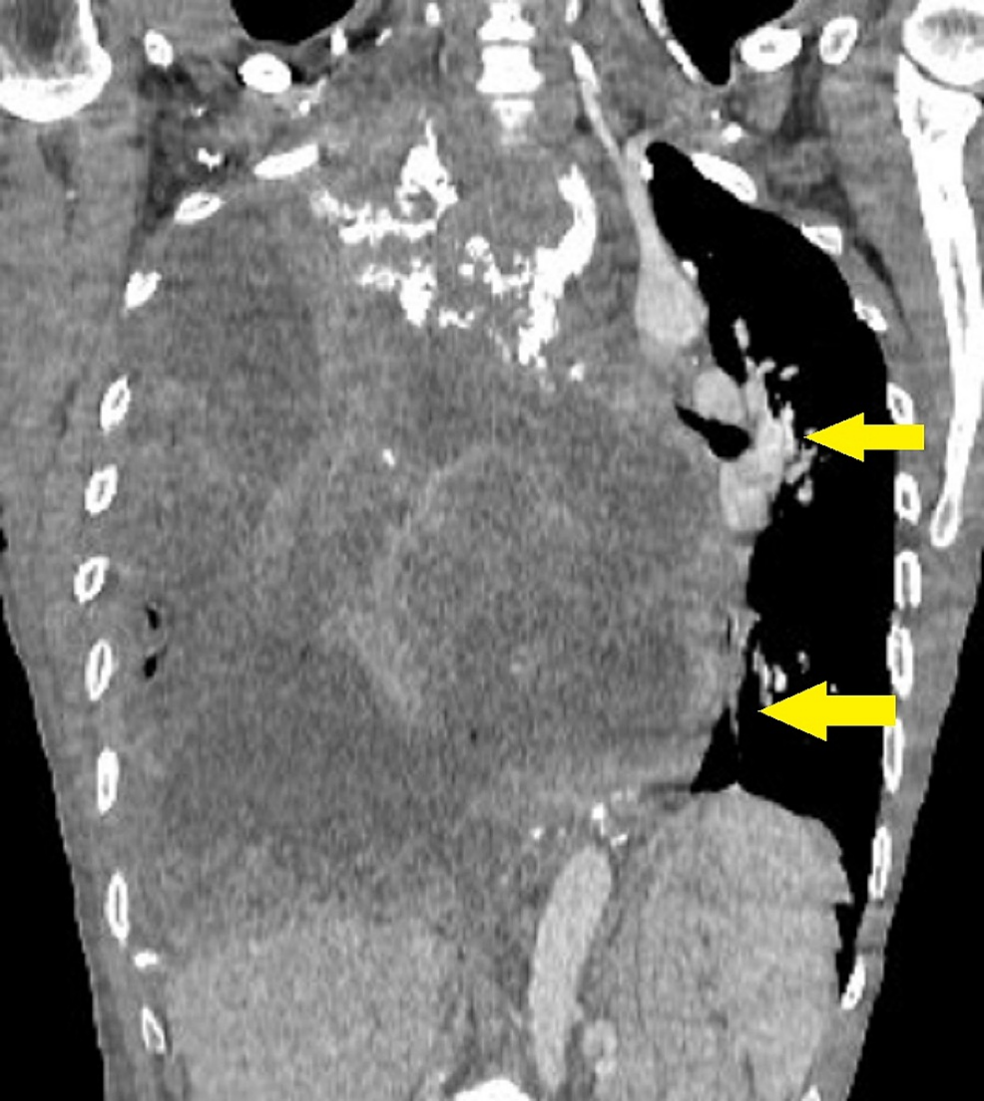
A coronal plane of the CT scan with injection of intravenous contrast showing the tumor occupating all the right hemithorax (arrows showing the deviation of the mediastinum).

**Figure 2:**
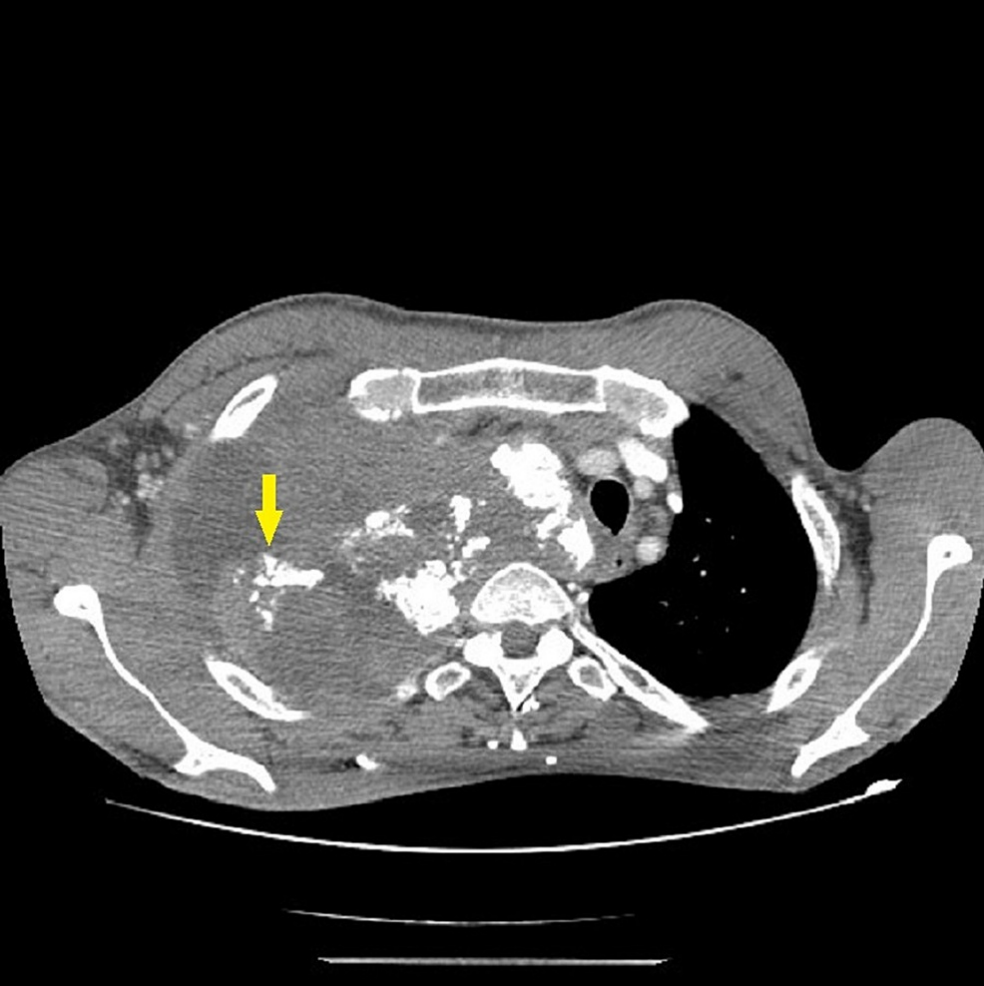
CT scan showing the tumor containing calcifications and invading of mediastinum

A 41-year-old man with a history of active smoking (20 packs/year) was admitted to the thoracic surgery department for the assessment of a complete white-out of the right hemithorax revealed by the chest x-ray. The thoracic CT scan showed a heterogeneous tissular process occupying the right pulmonary hemithorax (Figure 1), containing calcifications and invading and pushing the mediastinum towards the contralateral side (Figure 2).

Fibroscopy with bronchial biopsy showed a respiratory-type mucosa without abnormality. A biopsy of the right basi-thoracic pleural thickening after ultrasound detection came back negative. A trans-parietal biopsy of the pulmonary process was performed with an 18 G needle equipped with a coaxial system making six passages. The histological samples were compatible with chondrosarcoma. Cerebral and cervical-thoracic-abdominal-pelvic CT scans as part of the extension evaluation did not reveal any secondary distant localizations.

## Discussion

Rare lung tumors are defined as tumors of unusual histopathology in the lung parenchyma. Together, these tumors represent less than 1% of primary lung tumors, while encompassing more than 100 different histologic, clinical, radiologic, and evolutionary entities. Rare lung tumors arise from orthotopic, heterotopic, or hematopoietic tissue. Some histopathological subtypes are specific to the lung, while others are rare in the lung and correspond to tumors more frequent in other locations [1].

Pulmonary chondrosarcomas present as enlarged, lobulated, or cystic calcified masses with chondroid architecture. The histopathological examination should primarily confirm malignancy. The determination of the histopathological grade is important and follows the system of the French Federation of Anti-Cancer Centers, based on the mitotic index, the extent of necrosis, and tumor differentiation [2].

Careful histologic evaluation is also important because other lung lesions may be problematic, especially on small biopsies. For example, Smith et al., in 1960, reported the case of a 53-year-old woman who was initially diagnosed with a benign lung tumor and small biopsy samples were analyzed, the evolution of the clinical condition of the patient led to a reevaluation of the diagnosis and the conduction of a second biopsy which this time concluded to primary chondrosarcoma of the lung [3].

Chondrosarcoma is characterized by a masculine predominance. The average age, generally higher than that of patients with usual sarcoma, in most cases, is between 50 years and 70 years [4,5]. Symptoms depend mainly on the size and location of the lesion, are aspecific (cough, dyspnea, hemoptysis). Imaging shows a rather large mass (diameter between 4 cm and 25 cm), well limited, heterogeneous due to the presence of calcifications and cystic, necrotic, and/or hemorrhagic elements [6-8].

The treatment of pulmonary sarcoma is based primarily on surgery which, given the limited nature of these tumors, is associated with complete resection rates of over 80% [4,5,8]. Some teams recommend adjuvant chemotherapy [9]. Adjuvant radiotherapy is discussed in cases of high-grade lesions (80% of cases) or incomplete resection [10]. In the case of an unresectable tumor, treatment consists of chemoradiotherapy. Excision without ganglion curage but with an extemporaneous examination of the resection margins is recommended in the case of sarcoma [5,11].

The prognosis depends mainly on the ability to obtain a complete resection (50% to 80% of cases in published surgical series), which, combined with a low tumor grade, is the best prognostic factor for overall survival and recurrence-free survival [8,11]. The five-year survival rate, which is between 40% and 50%, is better in cases of chondrosarcoma [12]. On the contrary, the survival of osteosarcomas, which are rarely resectable, is less than 10% [13].

## Conclusions

Primary chondrosarcomas of the lung are rare. The primary origin of the lung is difficult to confirm and requires a careful evaluation of the clinical history as the lung is a common site of metastasis from different types of tumors, as well as careful histological analysis.

The treatment is mainly based on surgical resection, which when complete is a factor of good prognosis in combination with low histological grade. The data on chondrosarcomas remain very poor, and that also concerns other rare lung tumors, and observations like ours can contribute effectively in enhancing the scientific aspects of the pathology.
